# Effectiveness of intra-cardiac lidocaine and intra-amniotic digoxin at inducing fetal demise before second trimester abortion past 20 weeks at a tertiary Hospital in Ethiopia: A retrospective review

**DOI:** 10.1016/j.conx.2022.100082

**Published:** 2022-07-31

**Authors:** Abraham Fessehaye Sium, Tesfaye H. Tufa, Jaclyn M. Grentzer, Sarah Prager

**Affiliations:** a St. Paul's Hospital Millennium Medical College, Department of Obstetrics and Gynecology, St. Paul's Hospital Addis Ababa, Addis Ababa, Ethiopia; b Family Planning Division, Department of Obstetrics and Gynecology, UW Medicine, Seattle, United States

**Keywords:** Fetal demise, Feticide, Medication abortion, Second trimester abortion

## Abstract

**Background:**

Current literature recommends inducing fetal demise prior to second trimester medication abortion beyond 20 weeks of gestation. There is inadequate literature regarding the practice and effectiveness of this procedure in low-income countries, such as Ethiopia. This study aimed at documenting the effectiveness of intra-cardiac lidocaine and intra-amniotic digoxin at inducing fetal demise before second trimester medication abortion in an Ethiopian setting.

**Methods:**

This is a retrospective chart review conducted at St. Paul's Hospital Millennium Medical College, in Ethiopia. A total of 65 cases of feticide administration before 2^nd^ trimester medication abortion between 20 and 28 weeks of gestation (From April 1, 2021 to September 30, 2021) were reviewed. The primary outcome was cessation of fetal cardiac activity the day after the first feticide injection. Data were extracted by reviewing maternal charts using a data extraction tool prepared in English. Data were analyzed using SPSS version 23. Simple descriptive statistics were used to analyze baseline characteristics and fetal demise outcomes. Results were presented in percentages and frequencies.

**Results:**

More than three quarters of the feticide injections were with intra-amniotic digoxin, while the rest (24.6%, 16/65) were with intra-cardiac lidocaine. Injection of digoxin or lidocaine was effective at inducing fetal demise the day after administration in 92.3% (60/65) of the cases. Intracardiac lidocaine administration was 100% (16/16) effective at inducing fetal demise within the day after the injection while the effectiveness of digoxin within the same period was 89.8%.

**Conclusion:**

In this study, both intra-amniotic digoxin and intra-cardiac lidocaine were effective at inducing fetal demise, which is in support of findings from similar previous studies.

**Implications:**

In an Ethiopian setting, both intra-amniotic digoxin and intra-cardiac lidocaine injections are effective at inducing fetal demise before second trimester abortion beyond 20 weeks of gestation within the next day after feticide administration.

## 1 Introduction

The reasons for inducing fetal demise prior to second trimester abortion are fears of legal retaliation; comfort of the patient, provider and/or other involved health care workers; the belief that dilation and evacuation (D&E) will be easier and faster; to avoid transient fetal survival after medical induction; and to avoid extramural delivery with signs of life [1], [2], [3], [4], [5]. Among these reasons, avoidance of transient fetal survival after medication abortion and extramural delivery with signs of life are the most important reasons in the Ethiopian setting, where medication abortion is the most frequent method of later abortion. Pharmacologic feticide via injection of an agent into the amniotic fluid, fetal heart or umbilical vein is commonly utilized in contemporary obstetrics [6].

World Health Organization (WHO) abortion guidelines recommend consideration of fetal demise in medical termination of pregnancy above 20 weeks of gestation [7]. Inducing fetal demise before the second-trimester medication abortion can help circumvent the social, psychological and legal problems that face the team of caregivers, and the client undergoing abortion [8,9]. Moreover, there is an emerging evidence that beyond achieving negative fetal heartbeat, feticide administration before second trimester medication abortion shortens the induction to expulsion interval. A recent study from Turkey concluded that feticide procedure significantly shortens the induction to expulsion interval [10]. Currently, intra-fetal or intra-amniotic digoxin and intra-cardiac potassium chloride (KCl) are the pharmacologic agents most often used to induce fetal demise [11]. Intra-cardiac lidocaine is a pharmacologic agent also reported to successfully induce fetal demise [12,13].

Although induction of fetal demise is well studied in high-income countries,  there is inadequate literature regarding this topic in low-income countries. The practice of inducing fetal demise in low- and middle-income countries may be affected by resource constraints, unavailability of family planning specialist physicians, and patient and provider sociocultural norms and religious beliefs. Studying this practice in resource-constrained settings may help improve evidence-based practice in similar settings elsewhere. In our setting (St. Paul's Hospital Millennium Medical College in Addis Ababa, Ethiopia), patients who present for medication abortion in second trimester beyound 20 weeks are routinely provided with feticide administration before abortion. In the setting, resident physicians provide intraamniotic digoxin under the supervision of family planning fellows. Intraamniotic lidocaine is provided by family planning fellows and specialists only. This paper seeks to document induction of fetal demise in our setting.

## 2 Methods

This is a retrospective chart-review study conducted at St. Paul's Hospital Millennium Medical College (SPHMMC), in Addis Ababa, Ethiopia. A total of 65 cases of induction of fetal demise before second trimester medication abortion past 20 weeks of gestation, over 6 months period (April 1, 2021 to September 30, 2021) at SPHMMC were reviewed. SPHMMC is one of the leading tertiary Hospitals in Ethiopia and a national family planning excellence center. MICHU clinic is an organized sexual and reproductive health service center at SPHMMC, where family planning and comprehensive abortion care is delivered as a major health service pillar. At the clinic, injection of intra-amniotic digoxin or intra-cardiac lidocaine to induce fetal demise before second-trimester medication abortion. Resident physicians provide intraamniotic digoxin under the supervision of family planning fellows while intraamniotic lidocaine is provided by family planning fellows and specialists only.

Data on reproductive characteristics (age, gestational age, gravidity-parity…etc.), fetal demise characteristics such as feticide drug, level of care provider who administered feticide, and outcomes (frequency of fetal heart beat session after feticide injection and doses of misoprostol provided to complete medication abortion) were retrospectively collected. Data were extracted by reviewing maternal charts using a data extraction tool prepared in English. The inclusion criterion was medication abortion between 20 and 28 gestational weeks in which digoxin or lidocaine was injected to induce fetal demise. Charts were excluded if data on feticide agent administration characteristics and outcomes were incomplete.

The main outcome studied was the time from administration of feticide until negative fetal heartbeat (FHB) was detected. Negative FHB was required before initiation of misoprostol. On Day 1, in the outpatient setting, patients receive either lidocaine or digoxin injection along with mifepristone as part of the combination medication abortion regimen. Most patients receive intra-amniotic digoxin 1 mg transabdominally under ultrasound guidance. For those who cannot afford digoxin, lidocaine, which is provided free of charge, is used. For those receiving lidocaine, intracardiac injection is given transabdominally under ultrasound guidance, using either 20 ml of 1% lidocaine or 10 ml of 2% lidocaine and FHB is monitored 10 minutes after in injection to check whether cessation of fetal cardiac activity has occured.

Patients return on Day 2 in to check fetal cardiac activity. Those with documented absence of fetal cardiac activity are admitted for misoprostol induction (400 µg every 3 hrs sublingual for cases at less than 24 weeks of gestation and 200 µg for gestational age at 24 to 28 wks). Those who still have fetal cardiac activity are discharged to return on day 3. On day 3, those with cessation of fetal cardiac activity begin induction with misoprostol, while those who still have detectable cardiac activity have a second feticidal injection. On day 4, if still fetal heartbeat is detected positive, they take another dose of feticide injection.

We studied the frequency of negative FHB on day 2 (within 28 h hrs after 1st feticide injection), within day 3 (28–48 hrs after 1st feticide injection) and day 4 (>48 hrs after 1st feticide injection). These time periods correspond to proceeding with medication abortion on the planned date, a delay of one day before starting abortion, and a delay of more than one day in initiating the medication abortion. Negative FHB on day 2 was considered as an optimal clinical outcome and studied as a primary outcome of this study. This is in accordance to the standard about the starting of misoprostol during second trimester medication abortion, which is on day 2 or 24 hrs after administration of mifepristone. Achievement of this outcome was used to measure effectiveness of feticide drug.

Data were analyzed using SPSS version 23. Simple descriptive statistics were used to analyze reproductive characteristics, feticide admnistration characteristics, and fetal demise outcomes. Results were presented in percentages and frequencies. Formal letter of Ethical clearance for the study was obtained from IRB at SPHMMC.

## 3 Results

A total of 77 charts of cases of feticide injection before second trimester medication abortion were identified, and 65 cases were included in the final analysis after 12 charts were excluded for poor documentation of feticide administration time, feticide procedure provider, and other important reproductive characteristics. Out of those 65 cases, three quarters of the injections (49/65) provided were intra-amniotic digoxin (Table 1). the majority (72.3%, 47/65) of the subjects were primigravida. The calculated mean gestational age is 22.4 (20–26.6), with 29 (44.6%) of them being at gestational age greater or equal to 24 week. The mean maternal age is 22 (12–35). The most common indication for safe abortion among all the cases was maternal health, representing 59.4% (38/65) of the cases, followed by rape, accounting for 31.3% (20/65) of the cases.

**Table 1:** Baseline Characteristics

Variable	Category	Feticide used type					
		Intra-amniotic Digoxin (*N* = 49, 75.4%)		Intra-cardiac Lidocaine (*N* = 16, 24.6%)		Total (*N* = 65)	
		*n*	%	*n*	%	*n*	%
Gravidity	Primigravida	33	67.3%	14	87.5%	47	72.3%
	Multigravida	16	32.7%	2	12.5%	18	27.7%
Placenta position	Anterior	10	20.4%	3	18.8%	13	20.0%
	Posterior	4	8.2%	4	25.0%	8	12.3%
	Fundal	35	71.4%	9	56.3%	44	67.7%
Indication for abortion	Rape	16	32.7%	4	26.7%	20	31.3%
	Incest	1	2.0%	1	6.7%	2	3.1%
	Maternal health	29	59.2%	9	60.0%	38	59.4%
	Congenital anomaly (non-CNS)	3	6.1%	1	6.7%	4	6.3%
Age	Mean	23		22		22 (12–35)	
Gestational by 2nd trimester USG	Mean	22.6		21.7		22.4 (20–26.6)	
	≥24 weeks	22	45%	7	44%	29	44.6%

Most of the injections (69.2%, 45/65) were administered by obstetrics and gynecology residents (Table 2). Residents administered 91.8% (45/49) of the intra-amniotic digoxin injections, and none of the lidocaine injections. Intra-cardiac lidocaine was administered by the family planning fellows three quarters of the time, with the remainder provided by a family planning specialist.

**Table 2:** Feticide Administration Outcomes

Variable	Category	Feticide used Type						
		Intra-amniotic Digoxin (*N* = 49)		Intra-cardiac Lidocaine (*N* = 16)		Total		*p*-value
		*n*	%	*n*	%	*n*	%	
Feticide administered by	Resident	45	91.8%	0^1^	0.0%	45	69.2%	>0.05
	FP Fellow	3	6.1%	12	75.0%	15	23.1%	<0.05
	FP Specialist	1	2.0%	4	25.0%	5	7.7%	<0.05
Feticide administration to negative FHB interval in hours	Within 24-28 h (Day2)	44	89.8%	16	100.0%	60	92.3%	>0.05
	>28 ≤ 48 h (Day3)	3	6.1%	0	0.0%	3	4.6%	>0.05
	>48 h (Day 4)	2	4.1%	0	0.0%	2	3.1%	>0.05
Doses of misoprostol required	Mean	4		7		5		<0.05

Feticide was effective at inducing negative fetal heartbeat on day 2 (within 28 hrs after administration) in 92.3% (60/65) of the cases. Intracardiac lidocaine administration was 100% (16/16) effective at inducing negative fetal heartbeat by day 2 while the effectiveness of digoxin within the same time period was 89.8% (44/49). In three digoxin cases FHB became negative on day 3 (beyond 28 hrs but less than 48 hrs), and in other two similar cases a repeat dose of digoxin was required when FHB was still present on day 3 and negative fetal heart beat was detected on day 4 (beyond 48 hrs after initial feticide administration). The mean total dose of misoprostol required to complete the medication abortion was 5 doses. For patients who received digoxin, the mean was 4 doses, and for those who received lidocaine, the mean was 7 doses, which is statistically significant (*p*-value < 0.05).

## 4 Discussion

In this study, the effectiveness of either intra-amniotic digoxin or intra-cardiac lidocaine at inducing on the following day (day 2) after injection, was 92.3%. This finding is consistent with findings from previous similar studies. Sharvit et al. reported an effectiveness of 93.2% achieving negative fetal heartbeat 24 hrs after digoxin administration in his 2019 cohort study of 59 cases [14]. Recently, Tufa et al. found an 88.9% effectiveness of digoxin, administered a day prior to the start of medication abortion (within 24 hrs), at inducing fetal demise, in his 2020 review of 49 cases second trimester medication abortion [15]. Earlier, a randomized controlled trial of 134 cases of intra-amniotic digoxin administration one day before starting D&E procedures reported a success rate of 82.5%, which is lower than in our finding [16]. A prospective study of 26 cases of transvaginal intraamniotic digoxin, provided a day prior to the start of D&E found a success rate of 92. 4%, which comparable to our finding [17].

According to the literature, the utility of feticide for shortening the induction-to-expulsion interval during medical abortions is unclear. A 2019 study done in Turkey by Akkurt and his colleagues compared 56 cases of medication abortion at 17 and 28 weeks' gestation who received feticide to matched controls retrospectively. The study found that induction to expulsion interval was shorter in second trimester medication abortion cases who received feticide (900 ± 233 vs 1198 ± 375 min in those who didn't take feticide, *p* = 0.001) and prolonged medical abortion (induction to expulsion time >48 h) was less common in the feticide group (2% vs 6% in those who didn't receive feticide injection, *p* = 0.03). [10]. In our study, we found that more doses of misoprostol were required to complete the medication abortion for those subjects who received lidocaine as compared to digoxin. Although it is difficult to draw a cause-effect relationship given the retrospective nature of our study with a study design which is not ideal, this finding warrants further analytic study, as it suggests a potential benefit of intra-amniotic administration of digoxin at shortening induction to expulsion time.

Our study adds useful information to the existing literature on the practice of inducing fetal demise prior to medication abortion. Most importantly, it demonstrates the effectiveness of this procedure in setting of a low-income country, which may help improve evidence-based practice in similar settings elsewhere, where there may be scarcity of advanced abortion care providers (FP fellows or specialists). More than 90% of digoxin injections were completed by obstetrics and gynecology residents.

Limitations of this study include study design (descriptive study), which could be a potential source of bias. For example, sample size allocation in each group, selection bias, and the strength of the data analysis method used to measure and compare effectiveness. A randomized controlled trial would have been the ideal study design in this case. Retrospective data collection, not being able to analyze side effect profile of the feticide agents (data not collected), and lack of data on client acceptance of feticide options are the other limitations.

## 5 Conclusion

Both intra-amniotic digoxin and intra-cardiac lidocaine were found to be effective at inducing fetal demise within a clinically useful period of time, on the following day after feticide injection. This observation echoes the findings from previous similar studies. We recommend a further prospective study with a focus on comparing feticide side-effect profile; cause-effect relationship;client acceptance of feticide; and induction to expulsion time between these two common feticide drugs.

## Ethics approval and consent to participate

Ethical clearance was obtained from St. Paul's Hospital Millennium Medical College IRB

## Availability of data and materials

All data generated or analyzed during this study are included in this published article [and its supplementary information files]

## Authors' contributions

AFS, JG, and THT contributed research conceptualization, data collection and analysis. AFS, JG, and SP contributed data interpretation and manuscript write up. All authors reviewed the manuscript.

## Acknowledgments

Authors would like to acknowledge St. Paul's Hospital Millennium Medical College, Department of Obstetrics and Gynecology.
